# Supplementary material for: Splice-Junction-Based Mapping of Alternative Isoforms in the Human Proteome
Source: Cell Rep. Author manuscript; Available in PMC 2020 Jan 15. (PMC6961840; doi:10.1016/j.celrep.2019.11.026)
Supplement: 2 [file NIHMS1546469-supplement-2.zip › DF1/PXD009737/TestisLysC/TestisLysC_37_PHAX_KAIELLMETAEVEQNGGLFIMDIFYIENQK.pdf]

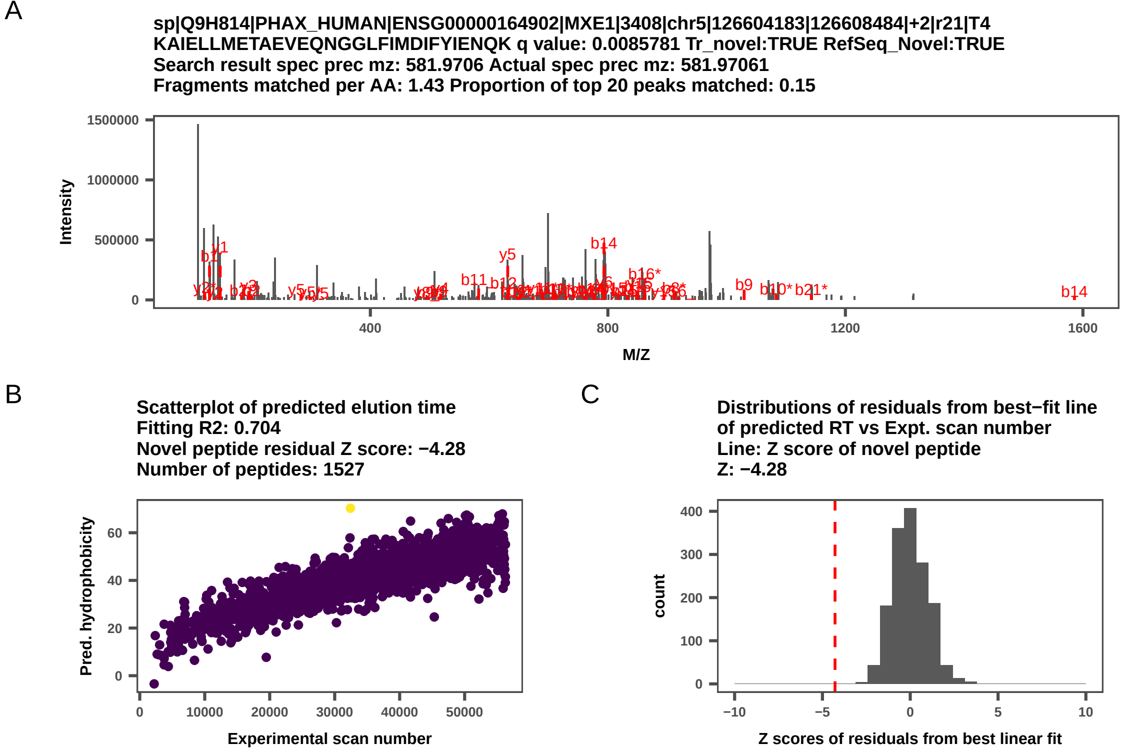
A
sp|Q9H814|PHAX_HUMAN|ENSG00000164902|MXE1|3408|chr5|126604183|126608484|+2|r21|T4
KAIELLMETAEVEQNGGLFIMDIFYIENQK q value: 0.0085781 Tr_novel:TRUE RefSeq_Novel:TRUE
Search result spec prec mz: 581.9706 Actual spec prec mz: 581.97061
Fragments matched per AA: 1.43 Proportion of top 20 peaks matched: 0.15
Intensity
1500000
1000000
500000
0
M/Z
400
800
1200
1600
y1
y5
b14
b11
b16*
b9
b21*
b14
B
Scatterplot of predicted elution time
Fitting R2: 0.704
Novel peptide residual Z score: −4.28
Number of peptides: 1527
Pred. hydrophobicity
60
40
20
0
Experimental scan number
0
10000
20000
30000
40000
50000
C
Distributions of residuals from best−fit line
of predicted RT vs Expt. scan number
Line: Z score of novel peptide
Z: −4.28
count
400
300
200
100
0
Z scores of residuals from best linear fit
−10
−5
0
5
10
